# Human causes of soil loss in rural karst environments: a case study of Guizhou, China

**DOI:** 10.1038/s41598-018-35808-3

**Published:** 2019-03-01

**Authors:** Longshan Zhao, Rui Hou

**Affiliations:** College of Forestry, Guizhou University, Guiyang, 550025 China

## Abstract

Rocky desertification induced by soil loss is a serious ecological problem in karst mountain areas. Lack of awareness in the local population of the need for soil conservation has led to intense human disturbance that has accelerated soil loss and in turn caused a high proportion of land in rural environments to undergo rocky desertification. In this review, we discuss five human-related causes that have accelerated soil loss in the rural karst mountainous areas of Guizhou Province, southwestern China. These causes include road erosion, house construction, steep slope cultivation, tourism development, and animal trampling. These activities destroy surface vegetation and increase the potential for soil loss through exposed swallow holes (karst fissures). In addition to the national development strategy of rural revitalization and countryside beautification already implemented in the western region, the human impacts on the rural environment must be addressed. We discuss some effective measures the government should adopt to control the various types of soil loss due to human activities. Our review and findings provide a better understanding of anthropogenic soil loss in karst rural environments and present information to raise people’s awareness of measures that are needed to protect the soil resources in this region.

## Introduction

Soil loss is a major environmental problem that results in land degradation, productivity decline, and ecosystem instability, all of which negatively affect the sustainability and healthy development of human society^1,2^. Soil loss has been studied across different regions, landforms, and landscapes, with a particular emphasis on the causes of soil loss and its influencing factors^3–6^. In general, soil loss has natural and/or human causes. The natural causes mostly include rainfall, vegetation, and terrain, while the human causes generally include activities that lead to adverse changes in regional vegetation, hydrologic processes, soil, and other environmental elements. Due to natural environmental differences, the types of human activities that can produce soil loss differ across regions, including rural areas^7,8^. Rural environments provide essential materials for both urban and rural populations, including the production of meat, grain, and water, and the characteristics and causes of soil loss in rural environments thus differ from those in environments with other types of land use^9,10^.

A number of studies have documented soil loss in rural environments. Gan *et al*. showed that^11^ the rate of soil loss due to unpaved roads, courtyards, and wastelands in three villages in the northern part of China’s Loess Plateau was 73.48, 20.82, and 68.73 Mg ha^−1^ annum^−1^, respectively, which is much higher than the rate of loss of bare agricultural soils, which is approximately 11.46 Mg ha^−1^ annum^−1^ ^12^. Cao *et al*. showed that^13^ water depth and the slope of road surfaces were the main factors affecting soil loss in the field and that the relationship of soil loss with water depth and slope could be described using a power function. Moreover, in some villages, these authors found that serious soil loss caused secondary disasters, such as landslides and road collapse. However, in almost all cases of serious soil loss leading to a secondary disaster, the cause was related to irrational and intensive human activities.

**Figure 1:**
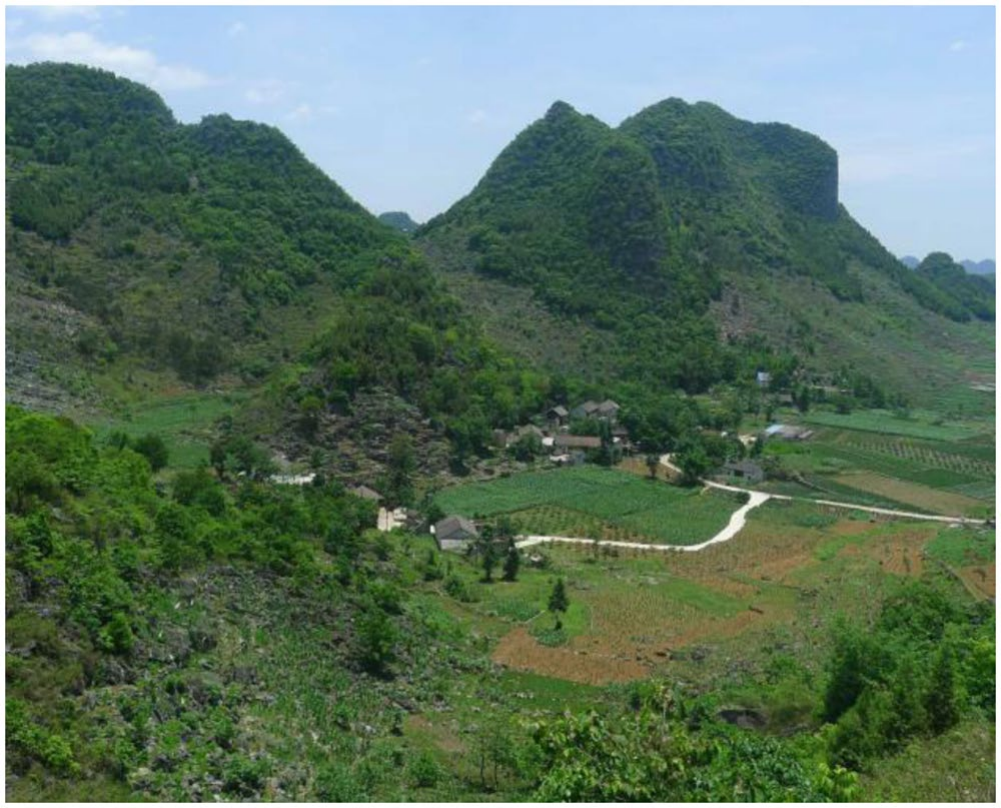
The rural landscape in karst mountain areas near Chuntianping village, Xingyi, Guizhou Province.

Guizhou Province is a mountainous area with a population of millions living in karst mountain villages, most of which are located on steep terrain with poor soil conditions^14^. In these villages, transportation infrastructure is poor, and production techniques are underdeveloped. Furthermore, these villages are overdependent on steep slope cultivation, grazing, and wood-based fuel, which has led to serious damage to the ecological environment, extensive soil loss, and widespread rocky desertification^15,16^ (Fig. 1). In rural areas, steep karst slopes are cut to build roads and are cut and filled for housing sites. In some villages, the materials used to construct houses and roads often draw on local resources, leading to the destruction of vegetation, which removes protection of the shallow soil on the karst slopes, particularly following rainfall^17^. Although soil loss is more serious in karst rural environments than in other environments, little information is available on its relationship with human activities; consequently, rural residents generally lack awareness of the effect of their actions on soil loss. In this paper, we analyse the characteristics and causes of soil loss in rural environments of karst mountain areas to provide a scientific reference for the study of soil loss in this region and to enhance people’s awareness of the necessity of soil protection measures.

## Study Area

**Figure 2:**
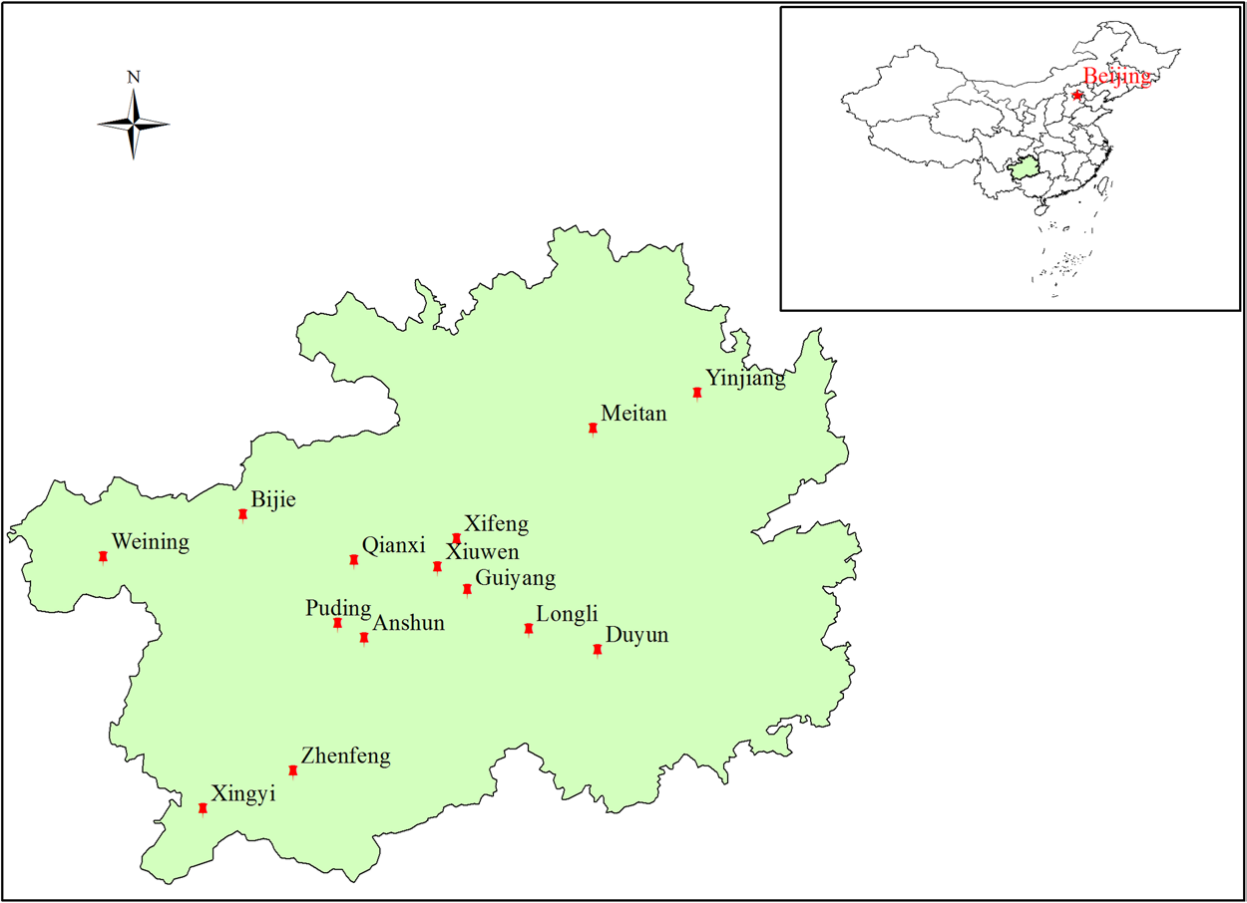
Location and distribution of the study area in China.

The study region is located in Guizhou Province, southwestern China (24°37′–29°13′N, 103°36′–109°35′E), and covers an area of approximately 17.6 × 10^6^ ha (Fig. 2). This region has a subtropical humid monsoon climate with an annual average temperature of approximately 15 °C. The annual rainfall ranges between 600 and 1400 mm, with most rainfall occurring from April to August. During the period when rainfall and temperatures are highest, the relative humidity averages over 80%. Based on 20 years of data from 99 weather stations throughout Guizhou, Zhou suggested that^18^ the erosional rainfall is 13.7 mm in Guizhou Province, and this value is greater than that observed in other regions in China (e.g., Loess Plateau, northeastern region). Based on data measured in karst runoff plots, Peng *et al*. noted that^19^ soil loss occurs on karst hillslopes only when rainfall is greater than 60 mm. The value of the mean rainfall erosivity factor (i.e., the R value in the revised universal soil loss equation (RUSLE) model) is 5299.8 MJ mm (hm^2^ h a)^−1^, and the spatial and temporal variations in this factor are significant^20^. The landform is plateau/mountain, and the terrain is high in the west and low in the east, with an average elevation of 1100 m. The soil is mainly composed of limestone from carbonate rocks, with a shallow depth, severe soil loss, and poor ecosystem stability. The area of rocky desertification is approximately 12.96 × 10^6^ ha, which is approximately 73.6% of the total land area of Guizhou Province^21^.

Due to the soil parent material and the subtropical humid monsoon climate, the soil coloration is black, yellow, and red, with a predominance of black in most areas of the karst mountain region^22^. The physical and chemical properties of the soil are listed in Table 1 ^22,23^.

**Table 1:** Physical and chemical properties of different lime soil types in the study area.

Soil type	Soil depth (cm)	pH	Soil bulk density (g cm^−3^)	Organic matter (%)	Total nitrogen (g kg^−1^)	Total phosphorus (g kg^−1^)	Total potassium (g kg^−1^)	Soil particle size (mm)		
								<0.002	0.002–0.02	>0.02
Black lime soil	0–5	7.09	0.99	5.08	3.12	0.54	27.35	36.04	14.39	49.57
	5–10	7.04	1.13	4.60	3.15	0.41	19.27	40.27	29.74	29.99
	10–20	7.02	1.11	4.48	2.99	0.69	21.73	43.56	35.62	20.82
Yellow lime soil	0–5	7.00	1.08	4.41	2.87	0.12	1.09	50.00	36.50	13.50
	5–10	6.99	1.22	5.73	3.53	0.19	5.76	59.50	27.70	12.80
	10–20	7.13	1.26	4.48	3.05	0.32	5.40	67.20	23.80	9.00
Red lime soil	0–5	7.46	1.28	0.38	0.34	0.19	13.77	41.50	38.60	19.90
	5–10	7.52	1.41	1.87	0.03	0.18	23.41	53.00	28.80	18.20
	10–20	7.32	1.53	2.45	0.39	0.19	17.42	19.90	50.20	29.90

### Characteristics of soil loss in karst mountain areas

Karst is a distinctive landform in which the topography is largely shaped by the dissolution of carbonate bedrock by water. Karst landforms mainly include peak-cluster depressions, gorges, and stone forests with steep slopes. The high gradient of karst landforms and high rainfall result in a potentially large soil loss rate in mountainous areas^24^; in contrast, the slow rate of soil formation by weathering of carbonate rocks and the shallowness of the soil both lead to a low ability of soil to counter the loss^25^. Additionally, karst soil is sufficiently loose and soil erodibility is sufficiently high to allow rapid detachment and loss by rainfall and runoff^11^. Vegetation is the only natural feature that protects the soils, but the vegetation of karst mountainous areas has limited soil conservation properties^14,26^. Karst mountain plants (calcicoles) thrive under calcium-rich conditions and display high drought sensitivity, causing them to have a slow growth rate and extreme sensitivity to environmental changes^27,28^. When these terrestrial ecosystems are affected by human interference, their vegetation tends to rapidly degenerate. In addition, due to the shallowness of the soil, the soil water-holding capacity is limited, and rainfall results in the loss of topsoil rich in organic matter, the destruction of soil structure, and a decline in soil fertility, which lead to vegetation degradation^29,30^. Due to these characteristics, karst mountain ecosystems tend to be unstable and have limited self-regulation, making recovery difficult once they are strongly disturbed by human activities. These factors provide the conditions for soil loss acceleration and rocky desertification.

**Figure 3:**
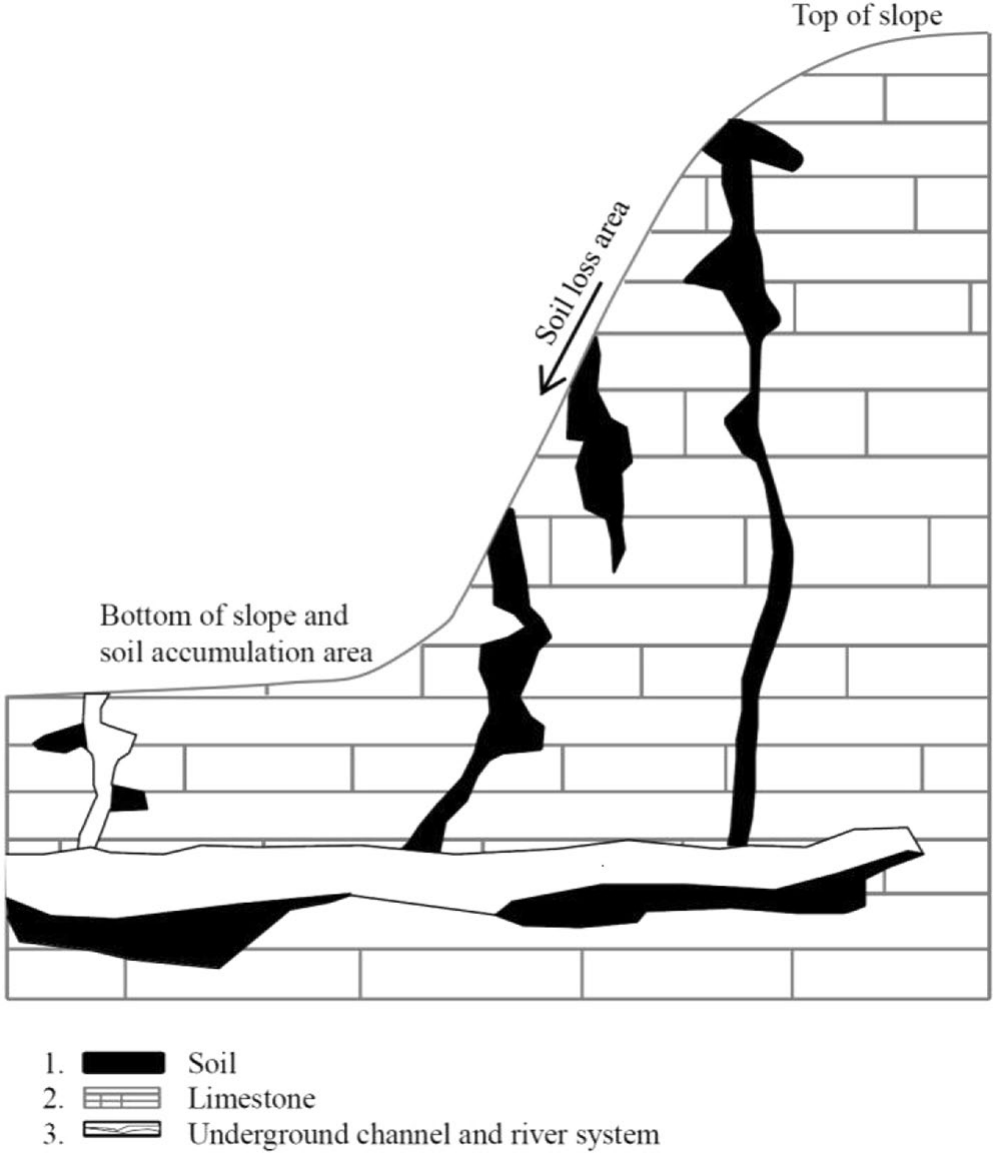
A schematic of the process by which soil is lost along a karst slope.

**Figure 4:**
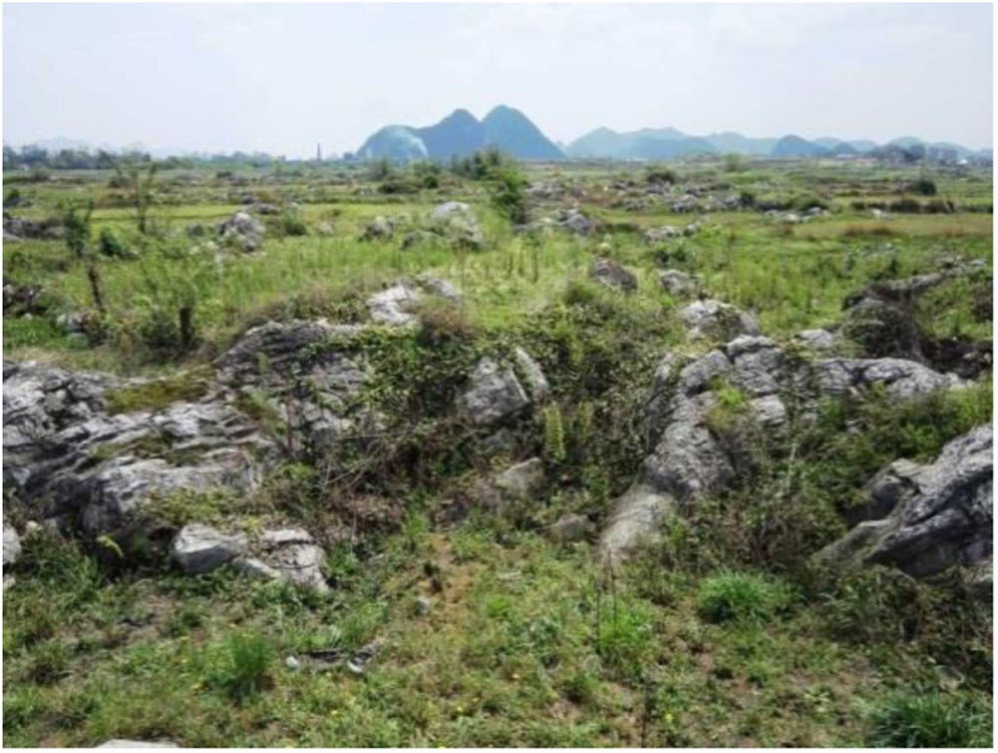
Rocky desertification of karst flat ground.

In addition to the phenomena described above, the long-term geological processes and chemical dissolution of carbonate rocks have resulted in surface streams, steep-sided gorges, swallow holes (fissures), underground streams and caves, and other geological structures in the aboveground and underground areas of karst landforms (Fig. 3). During rainfall, these special geological structures accelerate the leakage of surface water into subterranean areas^31^. Given the climate type and heavy, relatively concentrated rainfall, the surface soil is easily lost to erosion, with karst fissures, underground streams, and swallow holes providing channels and underground pathways. As a result, the surface soil layer gradually declines and becomes discontinuous, bedrock is gradually exposed aboveground, and the landscape typical of rocky desertification is formed. Rocky desertification is mainly distributed in the upper part of steep karst slopes. Surface soils not entering the underground channels are lost through surface runoff and accumulate at the bottom of the slope, forming a thick, fertile soil. During heavy rains, swallow holes at the bottom of the slope contribute to soil loss at the foot of the slope, and these soils enter underground streams. Therefore, rocky desertification also occurs on some mountain terraces of karst landscapes (Fig. 4).

Monitoring data have shown that^32^ the amount of soil loss in karst mountain areas can be up to 1.80 Mg ha^−1^. Findings of low soil loss are due to the loss of soil through underground karst fissures or swallow holes, leading to low values measured in surface runoff^33^. In contrast, when monitoring data show large quantities of soil loss, karst fissures and swallow holes are scarce, the loss of soil to underground features is low, and the loss to aboveground features is high^34^. However, due to monitoring limitations, there are no reliable data on the underground loss of soil. The amount of soil loss measured at different levels of rocky desertification shows that soil loss in areas without rocky desertification is high, with an average annual loss of 23.15 Mg ha^−1^ annum^−1^ ^19^. However, as the level of rocky desertification increases, the amount of soil loss decreases; in this scenario, the average annual soil loss is only 0.50 t Mg ha^−1^ annum^−1^ ^35^.

## Analysis of the Causes of Soil Loss in Rural Environments

Based on investigations of soil loss in karst mountain areas, vegetation coverage is higher (>50%) and the proportion of land showing rocky desertification is lower (<10%) in areas where there is little or no disturbance due to human activities^36^. In contrast, in areas inhabited by humans, especially near rural villages and sloping farmlands, the proportion of land subjected to rocky desertification is very high^31,37^, reflecting a large amount of soil loss, which is the only cause of rocky desertification in this region. Numerous studies have revealed many factors that affect the rate of soil loss^4,6,17^. In addition to the abovementioned natural causes and the special karst geology and geomorphology, irrational use and over-interference by humans are important causes of rocky desertification. Human impacts on soil in karst rural environments mainly result from the five following activities^38^.

### Roads

Four types of roads are common in karst rural environments, namely, rural roads, village internal roads, agricultural roads, and country lanes (footpaths). Areas occupied by roads account for 2.36–4.58% of the total area of rural environments^39^. Rural roads are the main channels through which residents communicate with other regions. The village internal roads are the channels used daily by rural residents. Agricultural roads are used for agricultural production, mainly for planting and land management, and their use differs according to season. During times of agricultural production, the high frequency of human and animal trampling results in high levels of soil loss; when farming activities cease for the season, there is less disturbance by human activities, road vegetation increases, and soil loss is low. Country lanes form through long-term trampling by humans and animals in the forest and grasslands near the village. Country lanes are narrow, have variable surface contours, and exhibit low vegetation coverage.

**Figure 5:**
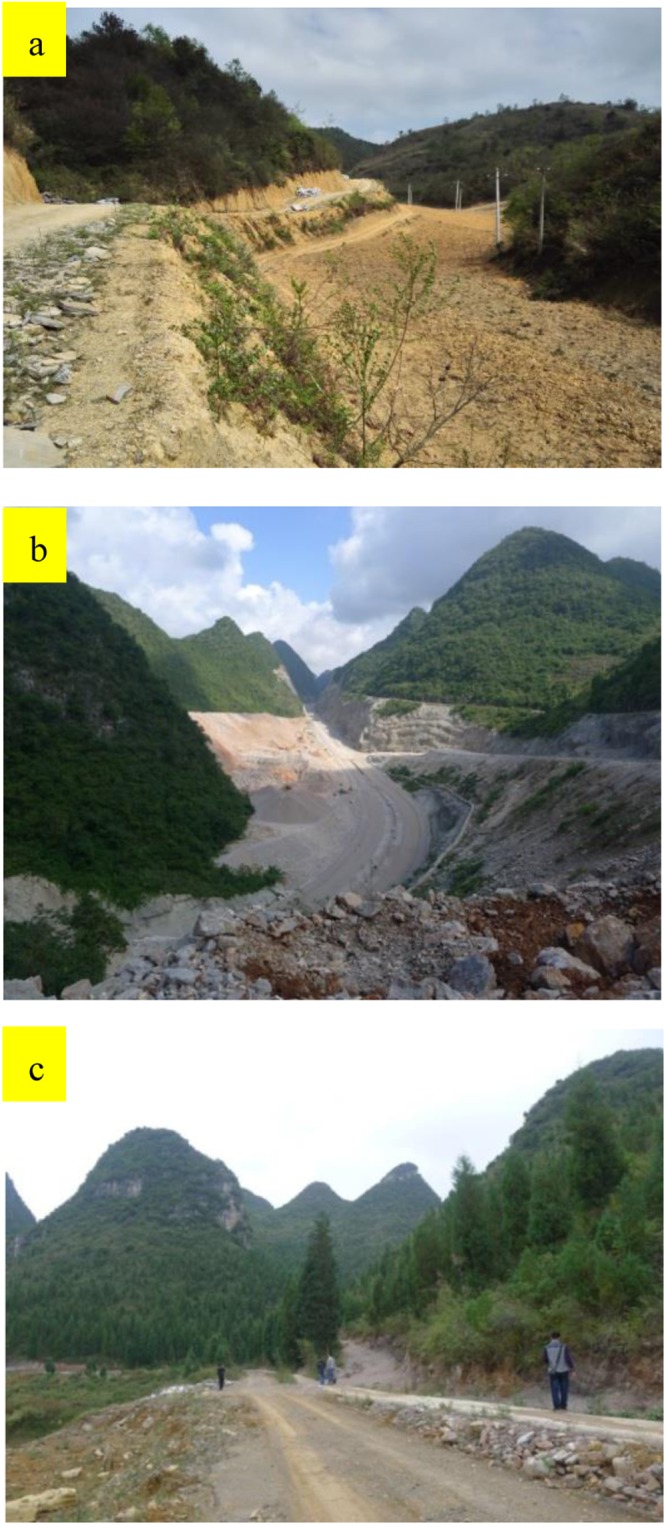
Rural road and rural road construction without soil conservation measures in karst mountainous areas.

Soil loss from roads stems from the direct impacts of road construction and from rainfall-induced losses from the road surface and the two sides of the roadbed (Fig. 5). Severe road erosion can cause the road to collapse and, in extreme cases, cause landslides. Due to the steep topography in karst rural environments, almost all rural roads have to be constructed by cutting into karst slopes (Fig. 5a). Road construction results in large exposed areas on both sides of the roadbed (i.e., cut-slope and fill-slope) (Fig. 5b,c), leading to new sources of soil loss^40^.

Soil loss from roads is also related to the structure of the roads. Rural roads are often paved, and diversion channels or ditches and small reservoirs are constructed on both sides of the road to collect runoff from the road surface and the upper hillslope. For these roads, soil loss is minimal. In contrast, roads without diversion channels lose soil. For mountainous unpaved roads, for example, the road surface is often below the terrain on both sides, and a gully subsequently forms in the middle of the road due to soil erosion^10^. Rural roads can also serve as special temporary corridors that are constructed as part of the infrastructure of new projects, such as wind farms and highway bridges, and these types of roads can also contribute to soil loss by disturbing vegetation cover and lead to considerable accumulation of disturbed soil^41^. Therefore, rural roads not only directly contribute to soil loss but also function as sediment transport channels and funnel soil from the upper slopes to drainage channels that enter a river or underground stream.

During the dry season, vehicular traffic and trampling by humans and animals generate a layer of loose soil on the surface of unpaved roads that is washed away through runoff during rainstorms. Soil loss from this loosened material can reach 0.82–20.27 Mg ha^−1^, and the amount increases depending on the thickness of the loosened soil and rainfall intensity^42^. The runoff coefficient^40^ of a single rainfall event can reach approximately 70% on an unpaved road without vegetation cover, with soil loss reaching 1.86 Mg ha^−1^. According to China’s classification standard of soil loss intensity (SL 190-2007), roads with such an intensity of erosion can be classified as strongly eroded^43,44^. Road erosion can therefore have serious consequences if not prevented.

### Housing excavation

**Figure 6:**
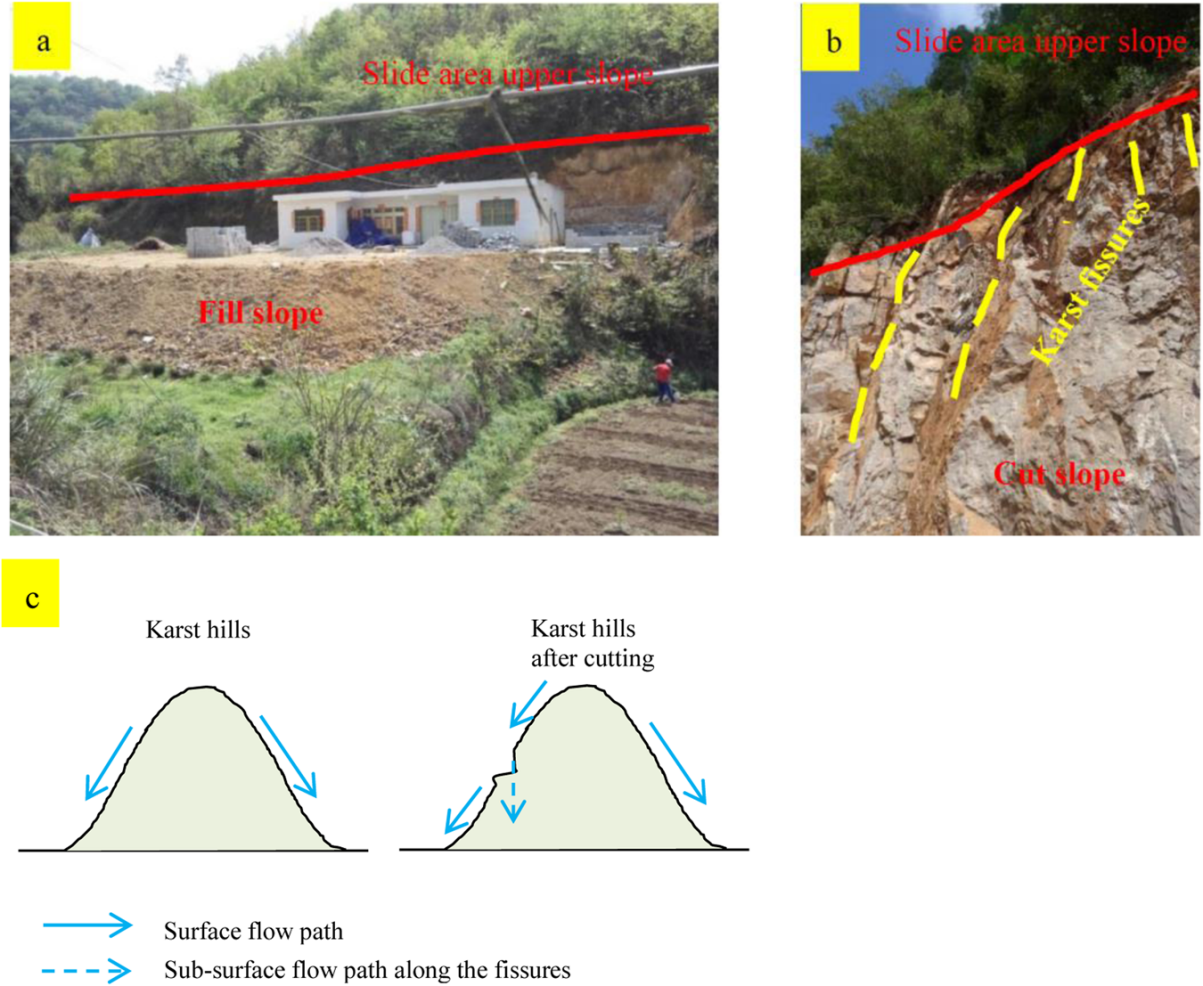
Soil loss due to housing site excavation and construction in karst mountains. (**a**,**b**) Cut and filled landforms due to house construction. (**c**) A schematic of the mechanism of soil loss due to excavation and construction.

Flat ground available for the construction of homes is minimal in rural karst mountain areas. In traditional village settlements, residents live in wooden lofts built on karst hillsides. The area of the lofts is small, and there is no need to cut the hillsides when they are built. Residents of these settlements are spread over a large area, and the impact of human activities on the surrounding environment, including soil loss, is low. However, with increasing socioeconomic development, residents improve their living conditions through the construction of houses and courtyards. Building a spacious homestead involves cutting and filling the steep karst hillslope (Fig. 6). The area of a traditional rural settlement in karst mountainous areas has generally been in the range of 0.01–0.03 km2, and land use has mainly included a house and livestock breeding stations^37^. Since approximately 2005, the land area of rural settlements has increased, especially for courtyards. Land use now not only includes basic living areas and livestock breeding stations but also land for warehouses, courtyards, and public services^45^. Rural areas also have the new function of nonagricultural production, which operates alongside the traditional functions of agricultural production and residential living^37,38^.

Measures to recover eroded slopes often are not taken promptly due to the lack of soil, slow recovery rate of vegetation, and lack of awareness of soil conservation measures. Housing sites generate runoff and can trigger surface erosion downslope. Soils at the upslope edge of the cut slope will slip during rainfall because the soil and vegetation stability under precipitation on the upper slope is lost. The original surface soil is lost during cutting into the karst slope, and fissures are exposed along the cut slope (Fig. 6b). During rainfall, these fissures, which link to other underground fissure paths to form a continuous subterranean network, function as channels and contribute to surface soil loss (Fig. 6c). In addition, the original steady state of the soil within the fissures is lost. Rainwater scouring along with gravity causes the soil stored in the fissures to loosen and move through the fissures. With the loss of soil stored in the fissures, soil can enter the fissures from above, thus increasing the total surface soil loss.

### Cultivation of steep slopes

Deforestation and reclamation of steep slopes for agriculture are primary causes of rocky desertification and soil loss in karst rural environments. These activities cover a wide area throughout karst mountains but particularly in Guizhou Province and are principally a result of population growth. The population of Guizhou Province has increased by 24.82% since the 1990s, and the agricultural population accounts for 70% of the total population^46^. Approximately 64% of the agricultural population still lives in rural karst mountainous areas^46^. Because of the growing population and limited available land, residents have reclaimed woodland and grassland near their villages to plant grains (usually maize) for food. With the destruction of forest and ground vegetation, the natural protection of the soil is lost, leading to soil erosion and reduced surface water storage. Over time, this soil damage progresses to rocky desertification. Because land reclamation for agriculture tends to be on steeper slopes, these areas suffer more soil loss than does surrounding land^47^. Guizhou Province has 638,400 ha of arable land with a slope >25°; this land accounts for 13.14% of the total arable land in the province, most of which is formed through forest reclamation^47^. In particular, the cities of Bijie and Zunyi have the largest proportion of land reclamation (approximately 20%), further exacerbating the rate of soil loss^47^.

**Figure 7:**
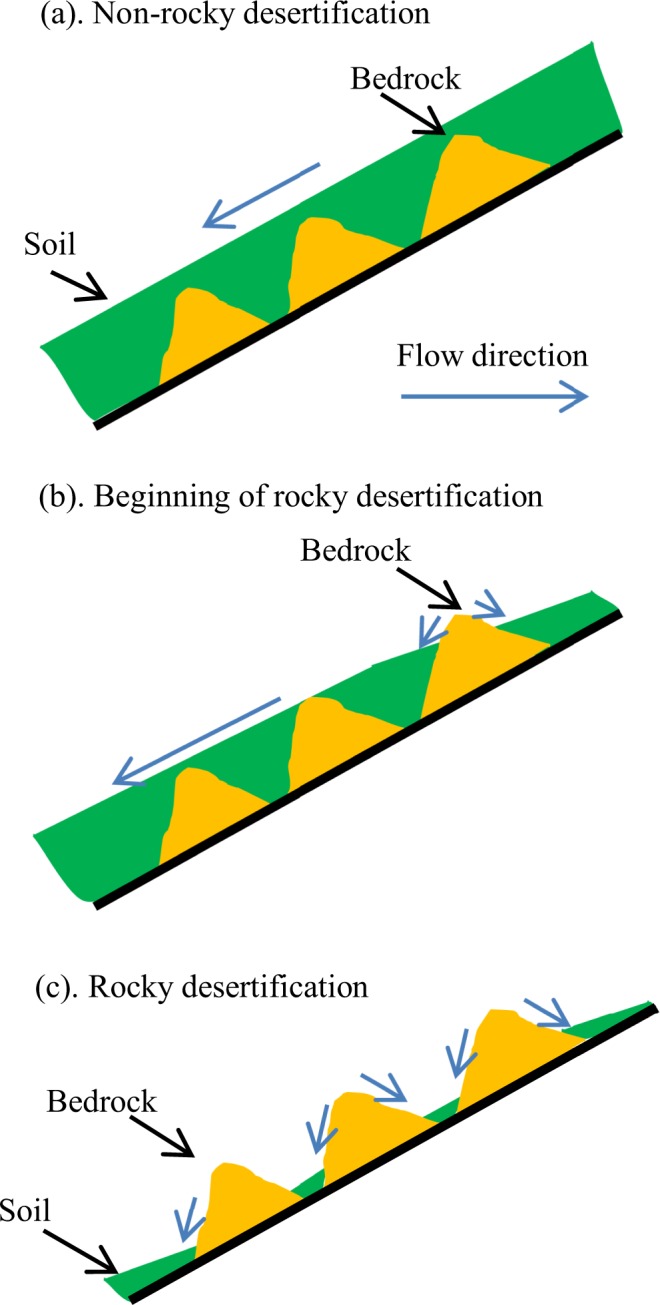
A schematic of the process of rocky desertification due to steep land cultivation in karst mountainous areas.

The process of rocky desertification can be summarized as follows^48^: steep slope reclamation → soil loss → bedrock exposure → rocky desertification (Fig. 7). Rocky desertification is an irreversible process, which means that once it occurs, soil quality and productivity will decline and never recover. The original steep slopes generally have high soil quality; however, planting time increases on reclaimed agricultural land, the spatial distribution of soil gradually changes from patchy to scattered patchy, and the area with soil available for farming is reduced. Low productivity dominates where rocky desertification has become severe. Because there are few single tracts of land in mountainous areas, the availability of land with soil suitable for local residents is reduced.

Features of soil loss differ between land subjected to rocky desertification and that without rocky desertification due to the influence of exposed bedrock. Exposed bedrock does not intercept rainfall and is not infiltrated by rainwater. The flow of rainwater follows the bedrock’s surface and increases surface soil runoff around rocks. Upslope soil is transported downslope until the flow velocity decreases at the slope bottom, where the slope gradient is shallower. The sediment is then gradually deposited, resulting in a thicker layer of higher-quality soil at the slope bottom. In addition, in areas with severe rocky desertification, swallow holes often develop^32^. With heavy rainfall and more runoff from upslope, the soil deposited at the bottom of the slope can be resuspended and transported into a deeper underground karst fissure or underground river.

### Tourism

Rural tourism in karst mountain areas is an important mode of poverty alleviation and economic development. More than 1000 villages in Guizhou Province depend on rural tourism and the leisure industry as part of their economy. With an annual average of more than 20 million tourists^48^ and an annual income of more than 30 billion yuan (ca. 4.8 billion USD), tourism has become a pillar industry in Guizhou Province and an important way for the area to promote the development of its rural industries, lift the rural poor out of poverty, beautify rural areas, and rejuvenate the countryside.

Over the last decade, land use patterns in rural environments have undergone major changes. Land use is no longer confined to traditional agricultural production and now focuses on diversified development. The development and construction of tourism resources will inevitably lead to changes in the original rural landscape and vegetation, which in turn will impact soil stability and soil loss. In some popular tourist areas, issues such as transportation and accommodation need to be addressed, and land development and infrastructure construction are required. Such projects will inevitably change the original landform and thus affect soil loss. The rural environments of karst mountainous areas have many natural landforms, such as peak forests, tiankengs (dolines), caves, and stone forests. To attract more tourists to traditional ethnic villages, local governments intend to undertake a number of new tourism projects combined with neighbouring natural landscapes. These activities will also have an impact on soil loss.

In addition, as the number of tourists increases, the demand for water will increase. Although karst villages have a subtropical monsoon climate with abundant rainfall, due to the development of karst fissures, precipitation is easily lost through underground rivers. Consequently, karst rural environments can experience severe water shortages in the autumn and winter. To maintain the quality of tourism year round, engineering measures for water allocation, such as reservoirs, ponds, and water pipelines, are needed. The construction of these measures will inevitably change the local hydrological processes, thereby impacting soil loss. Another impact will stem from outdoor tourism, such as mountain climbing, whereby trampling of the vegetation and ground will lead to soil loss. Thus, changes in the erosional environment due to construction activities and increased tourism are the main cause of soil loss associated with tourism and recreation.

### Animal trampling

Soil loss due to animal trampling in rural karst mountain environments mainly occurs through three types of action: feeding by livestock, pen production, and free-range production. To plough land for farming, most families raise one or two livestock; in general, such livestock include cattle or horses. In addition, because travel in this region is difficult, these livestock are also used to transport goods and materials on a daily basis. When not being used for farming, livestock are taken to grassland or uncultivated land to feed on grasses and are constrained with a rope to prevent them from feeding on nearby crops. The length of the rope varies and can be up to 100 metres, with one end connected to the ground and the other tied to the livestock. With this technique, the range of feeding is a circle, and the largest distance is the length of the rope.

Pen production is generally used for poultry and pigs. Animals are enclosed in a fence, with pen sizes ranging from less than 2 m^2^ for one pig to greater than 100 m^2^ for hundreds of chickens. In contrast, free-range animals can walk and feed freely over a much wider area compared with the first two types of animals and mainly eat weeds, worms, insects, and grasses; there is almost no artificial feeding under such practices.

These three types of animal trampling have a significant effect on soil loss but to different extents. Because the range of movement for cattle and horses is limited, the trampling intensity of surface soil is high, with many areas being trampled multiple times. As a result, grass is eaten, broken, or crushed. Moreover, the heavy weight of the livestock compacts the surface soil and leaves many footprints, which decrease soil porosity. During rainfall, the trampled ground has a low soil infiltration rate and easily generates surface runoff to trigger soil loss. In the case of pen production, a fence is generally erected around unvegetated land, and animals are kept in captivity. Soil loss due to pen production is similar to that due to road erosion. Animal trampling causes the formation of a soft soil surface layer that has high erodibility and easily results in soil loss on windy and rainy days. In contrast, free-range animals eat grass and forest litter, thereby reducing the amount of existing surface vegetation and decreasing its soil conservation function. In addition, pigs use their snouts to root for nuts around trees or to dig mud wallows. Poultry, however, usually peck at the soil to obtain weeds, worms, and insects. These behaviours can change the surface microrelief and hence increase soil loss. Before 2000, free-range grazing, mainly by goats, was common; however, this practice has been banned as policies to protect the ecological environment have been implemented by the state. Free-range grazing has been replaced by ecological breeding in which animals are grass-fed in captivity. Consequently, the impact of free-range grazing on soil loss has been reduced.

In karst mountainous areas, the shallow soil and trampling due to grazing combine to result in more environmental damage. Because of the steep slope of the terrain, surface soil particles are more prone to move downslope. As a result, animal trampling has a notable influence on the stability of the soil structure and surface vegetation and hence can greatly influence soil loss.

## Challenges and Opportunities for Soil Conservation

Rocky desertification is a common phenomenon worldwide due to the impact of human activities. For example, in the Mediterranean Basin and Dinaric karst region in Europe, cutting forest for agricultural production, overgrazing, farming on steep hillslopes and burning of forests are related activities that cause rocky desertification^30^. In Guizhou Province of China, the main cause of rocky desertification is human activity^49^. Therefore, limiting human disturbances to land is the most effective way to control rocky desertification.

In the 1980s, soil loss and rocky desertification in karst areas attracted the attention of China’s central government. In particular, a national project called “Development of the Western Regions” was initiated in 2001 to develop the economy of the western regions. At the same time, several major ecological conservation projects, including ecological migration, establishment of nature reserves, and the Grain for Green program, were also implemented to “tame” karst rocky desertification^50^. To some extent, those projects have played important roles in limiting the spread of rocky desertification and soil degradation. However, rocky desertification is a complex process and cannot be addressed in the short term. Limited knowledge of the mechanisms driving soil loss and rocky desertification, especially for soil loss through karst underground fissures and swallow holes, along with a lack of basic research restricts the sustainability of soil conservation efforts. The results from current soil and water conservation projects indicate that attention is mainly focused on preventing soil loss at the ground level, but little attention has been paid to soil loss through underground channels. This shortcoming raises questions about the usefulness of existing soil and water conservation projects and is resulting in a failure to limit the damage caused by construction and interference from human factors.

The impact of human factors on soil loss and rocky desertification is also related to the development of society. Over the last few decades, China has grown quickly. Local governments are trying to maintain relatively rapid economic growth and thus have reduced the strict oversight associated with environmental protection, especially in undeveloped places. The karst of Guizhou Province is located in the southeastern mountains of China, where economic development is poor. As a result, soil loss induced by human activities is not attracting the attention that it would in areas with a more advanced economy.

To address the lagging development in rural areas, in 2013, China’s central government mandated that the beauty of the rural countryside be promoted by protecting the ecological environment of rural areas and encouraging the development of sustainable agriculture, orchards, husbandry, and rural tourism to stimulate economic development. The Chinese government also put forward a strategy for rural revitalization in 2017. The main aims of this strategy are to improve living and traffic conditions, increase economic prosperity and the health of rural residents, and beautify rural environments. To achieve these aims, extensive infrastructure for roads, communication, water and sanitation facilities, and medical services are needed. Infrastructure construction would change the natural environment and impact soil loss. Therefore, soil conservation is a top future priority for this region.

In addition to the reasons mentioned above, all of which contribute to the serious loss of soil and rocky desertification of karst rural environments, the lack of effective supervision and management cannot be disregarded. Because the human causes of soil loss have been ignored in the past, scientific monitoring of soil loss in rural portions of karst areas has not taken place. Moving forward, the most significant steps to address soil loss in karst rural environments should be the following: (1) Take effective measures to prevent soil loss during development activities while also enhancing supervision and soil loss monitoring by implementing strict management systems^37^. These measures include changing traditional planting modes and implementing tillage techniques with soil and water conservation functions, such as contour ploughing, reservoir tillage and conservation tillage^16,51^. Currently, the corn-growing area in this region is very large and should be reduced. Most areas of Guizhou Province are characterized by large amounts of precipitation, which facilitates the development of ecological agriculture such as the pepper grass-goat and cattle-fruit-vegetable modes of production. Such development improves the productivity and economic benefits of land and in turn reduces the land needed for development by local residents^52,53^. Furthermore, on steep slopes (>25°), grazing, deforestation, agricultural production and house excavation should be prohibited, and such land should be used to plant water conservation forests^29,54^. To prevent soil and water loss through underground fissures due to excavation and construction, it is strongly recommended that the fissures on cut slopes be blocked immediately. (2) The government should increase its input by conducting studies on the processes and mechanisms of soil loss induced by human activities to provide a scientific reference for land or project managers and devise appropriate management systems. It is necessary to increase the areas in which technology is demonstrated and soil and water conservation of rocky desertification areas is promoted and to strengthen training of local residents in soil and water conservation techniques^21,37^. (3) Nature reserves should be created in rural areas with extensive rocky desertification to help minimize the factors contributing to soil loss. In nature reserves, building houses and other human activities that may cause soil loss without government permission should be forbidden^55,56^. Development and construction projects of all sizes in karst mountain areas should adopt science-based water and soil conservation schemes and eventually arrange water and soil conservation measures^24^. If we do not adopt effective ongoing measures to control soil loss, then any success we have had in the past in controlling rocky desertification will be wasted.

## Conclusions

Rocky desertification of karst landscapes is more serious in rural mountainous areas than in other areas due to the fragility of the ecological environment, lack of soil resources, extensive soil loss, and disturbance to the natural environment caused by human activities. In rural landscapes, human factors that mainly involve road construction, excavation for homesteads, steep slope reclamation for agriculture, rural tourism development, and grazing activities accelerate soil loss and increase the extent of rocky desertification. Human activities impact soil loss directly through physical disturbance and indirectly by changing the underlying properties of the soil, making it susceptible to loss with rainfall. Particular attention should be paid to the many underground subsurface fractures that were once stable. Once these features are excavated and the holes are exposed, the soil that is present in the holes will quickly be lost, accelerating the entry of surface soil from the surrounding area to fill the gap.

Our findings further suggest that as human activity destroys surface vegetation, it provides the conditions for soil and water loss. At the same time, underground holes and fissures induced by cut hillsides may increase underground erosion. Although the five human activities discussed above also occur in non-karst regions, they can have more serious consequences in karst mountainous areas due to their complicated landforms, small amount of soil and large amount of precipitation. Our review presents the most thorough examination of anthropogenic soil loss in karst rural environments to date and provides information to raise people’s awareness of measures that are needed to protect the soil resources in this region.
